# A Bibliometric Analysis of Literature on Prenatal Exposure to Air Pollution: 1994–2022

**DOI:** 10.3390/ijerph20043076

**Published:** 2023-02-09

**Authors:** Bukola G. Olutola, Paulina Phoobane

**Affiliations:** 1 School of Engineering, Science and Health, The Independent Institute of Education (IIEMSA), Roodepoort 1724, South Africa; 2 School of Information Technology, The Independent Institute of Education (IIEMSA), Roodepoort 1724, South Africa

**Keywords:** air pollution, prenatal exposure, intrauterine exposure, bibliometric review

## Abstract

Early life exposure to air pollutants during pregnancy is one of the leading causes of different health outcomes. However, few studies have provided an overview of this area of research. The aim of this study was to look at the key trends in the research on prenatal exposure to air pollution. Data were retrieved from Web of Science, and the search was conducted based on the paper title, abstract, and keywords. The relevant literature searched was from 1994 to 2022, and 952 English documents were obtained. Of the total documents, 438 documents were included in the review and 83% (*n* = 365) of the documents were journal articles. Type of document, annual distribution of publications, and distribution of prenatal exposure by countries were extracted. Co-authorship and keywords co-occurrence analyses were also carried out. Of all the countries that published in this field, the United States of America. had the highest number of publications, followed by China. Among the different health and environmental disciplines, 62% (*n* = 273) of papers came from environmental science. There were limited collaborations among researchers from different countries and institutions. In conclusion, there should be more collaboration among the researchers in this field regarding institutions, countries, and disciplines.

## 1. Introduction

The impacts of air pollution on health have attracted worldwide attention because of its big disease burden [1]. In 2017, air pollution was the fifth highest mortality risk factor globally and was associated with about 4.9 million deaths and 147 million years of healthy life lost [2]. Exposure to air pollutants during pregnancy can be harmful to children and later into adulthood because this is the phase of growth and development [3,4]. Many recent studies have analyzed the importance of exposure to air pollutants during intrauterine life and the possible repercussions of said exposure at birth, during childhood, and during adulthood [5].

The health effects of prenatal exposure to air pollution have been shown in numerous studies [3,6]. Hsin-Chien et al. [6] found that PM_10_ increased the risk of immune thrombocytopenia, while Su et al. found an association between PM_1_, PM_2.5_, PM_10_, and SO_2_ and adverse neurodevelopmental effects in the early life stage. Other health effects of prenatal exposure include respiratory problems [7], cardiovascular diseases [8], fetal macrosomia [9], and autism spectrum disorder/attention deficit/hyperactivity disorder [10].

One of the study designs that has been used to assess the impact of prenatal exposure to air pollution is a cohort study. It has been used to measure exposure to air pollution during pregnancy, and longitudinal follow-up has been used to analyze the outcomes for the fetuses at birth and following birth. Several cohort studies have been conducted to further elucidate the relationship between prenatal exposure and health outcomes [5,11]. However, there is a limitation on the studies that have determined the research status in the literature of fetus prenatal exposure to air pollution. A bibliometric review is an appropriate methodology to identify the volume and growth pattern of the literature focused on the health effects of prenatal exposure to air pollutants.

Therefore, we sought to look at the key trends in the research on prenatal exposure to air pollution.

## 2. Materials and Methods

The data used in this research study were retrieved from Web of Science (WoS). WoS is one of the popular databases used for almost all disciplines. These data were the bibliographic metadata of the papers on prenatal exposure to air pollution and their cited papers. The data were retrieved on 11 October 2022 with all-time span (1976–11 October 2022). There were no restrictions on document type or geographic scope. The search was performed at the topic level, where the search was conducted on the paper title, abstract, and keywords. An advanced search was used to minimize the retrieval of irrelevant papers. The following search string was used to retrieve the data:

TS = (“prenatal exposure” OR “intrauterine exposure”) AND TS = (“air pollution” OR “SO_2_” OR “NO_2_” OR “OZONE” OR “PM_2.5_” OR “PM_10_”)

**Figure 1:**
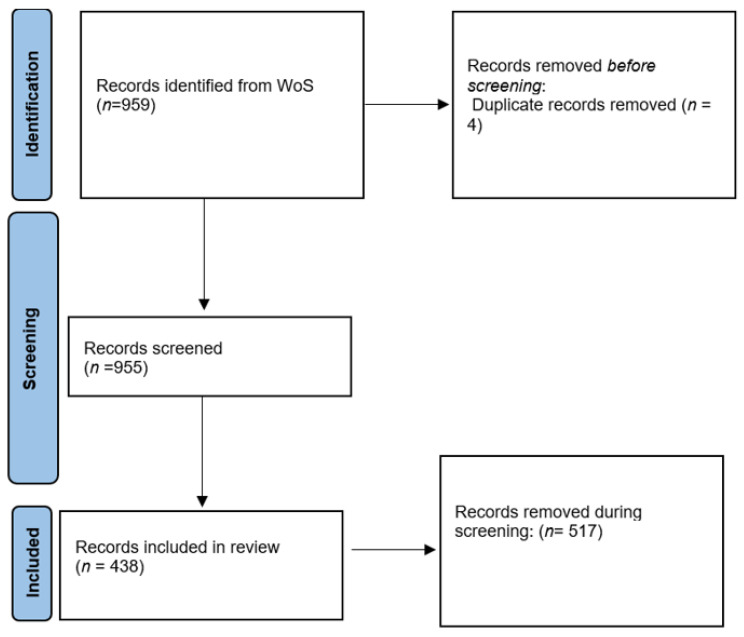
Process of identifying documents for study inclusion.

The search resulted in 959 papers. The data comprised information about the document, authors, content, citation, and funding. The document/paper information included the title and publication year, while author information included the author’s name and affiliation. The funding includes information such as the name of the funding organization and funding number. Due to the inherent drawbacks of retrieval techniques, the retrieved papers were reviewed and evaluated to improve the quality of the dataset. The screening was manually performed on the title, abstract, and, where necessary, at the content level. Only papers meeting the inclusion criterion were considered for further analysis. The inclusion criterion was that the papers should be on prenatal exposure to air pollution. During the manual screening of the papers, 4 duplicates were identified and removed. The bibliographic metadata of the papers considered for inclusion in this paper were downloaded as plain text (.txt). Figure 1 depicts the process of identifying papers for analysis in this research study.

The dataset that was considered for analysis comprised different types, as indicated in Table 1. The papers were mostly articles. Book chapters and collections were the least represented, with only one each. 

The cleaned data were then analyzed using VOSViewer version 1.8.18. VOSviewer is a rigorous software developed by professors Van Eck and Waltman from the Centre for Science and Technology Studies at the University of Leiden [12]. It has been used to execute bibliometrics analyses to describe the structure and evolution of a certain research field. Several research studies in various research fields have used VOSViewer to unpack the research trends, gaps, and opportunities [13,14,15]. We used VOSViewer to explore the research trends, gaps, and future research opportunities in the publications on prenatal exposure to air pollution through the following visualization maps: co-citation, keywords co-occurrence, and co-authorship maps.

## 3. Results

### 3.1. Prenatal Exposure to Air Pollution Research Trends

**Figure 2:**
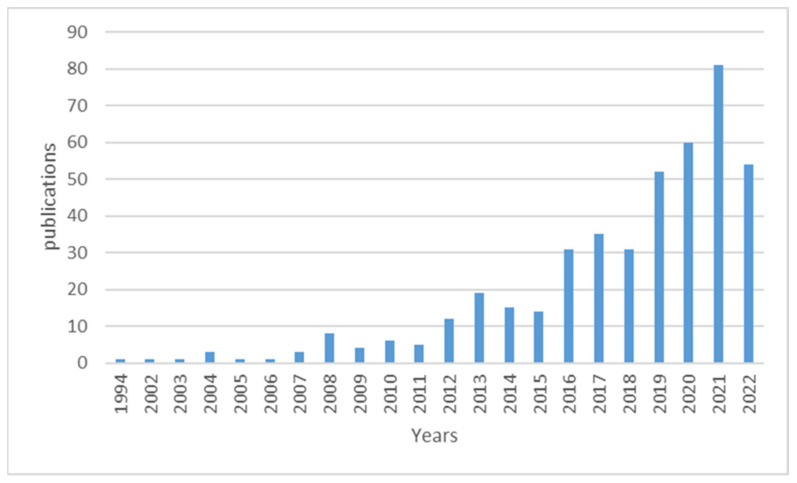
Annual distribution of publications on prenatal exposure to air pollution.

Figure 2 below shows the trend in global publications on prenatal exposure to air pollution from the year 1994 to 11 October 2022. It can be noted that the first publication on prenatal exposure to air pollution was published in 1994 as per the dataset from WoS. It can also be observed that there has been a steady, gradual growth in publications in this field. The year with the highest publications is 2021, followed by 2020 and 2022 with 81, 60, and 54 publications, respectively. The year 2021 also shows the highest increase rate of 4.8%. It can also be observed that there was a slight decrease in publications in 2022. This could be attributed to the fact that 2022 data were incomplete as the authors considered publications up to 11 October 2022. On the other hand, the years with the lowest publications are 1994, 2002, 2003, and 2005, with one publication each.

#### 3.1.1. Publications by Regions/Countries

**Figure 3:**
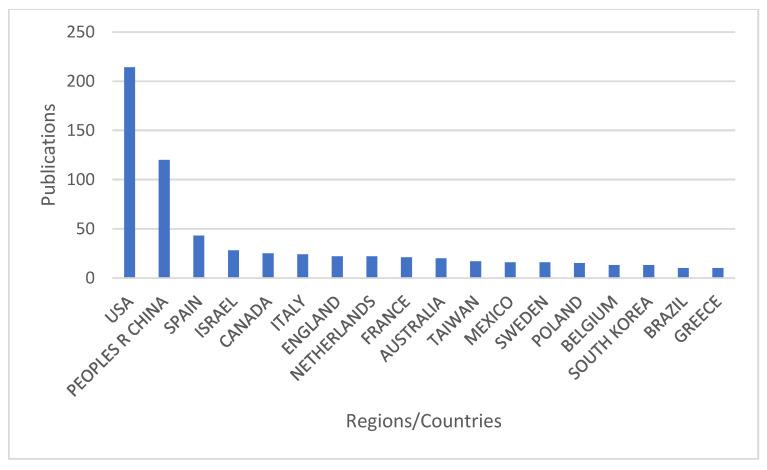
Annual distribution of prenatal exposure to air pollution publications by countries and territories.

Prenatal exposure to air pollution has attracted the attention of researchers globally. Figure 3 below illustrates the countries/regions that have contributed to the prenatal exposure to air pollution publications. Only the top 20 countries/regions are considered in the graph below. It can be observed that the leading country is the United States of America (USA), followed by the People’s Republic of China, Spain, Israel, and Canada. The USA had a publication total of 214, which amounted to 48.9% of the total publications on prenatal exposure to air pollution globally. On the other hand, the People’s Republic of China, Spain, Israel, and Canada had 120 (27.4%), 43 (9.8%), 28 (6.4%), and 25 (5.7%) publications, respectively. Europe was the most-represented continent in the top leading regions, followed by the America. Continents such as Africa were not present in the top leading territories.

#### 3.1.2. Contributions by Authors

**Figure 4:**
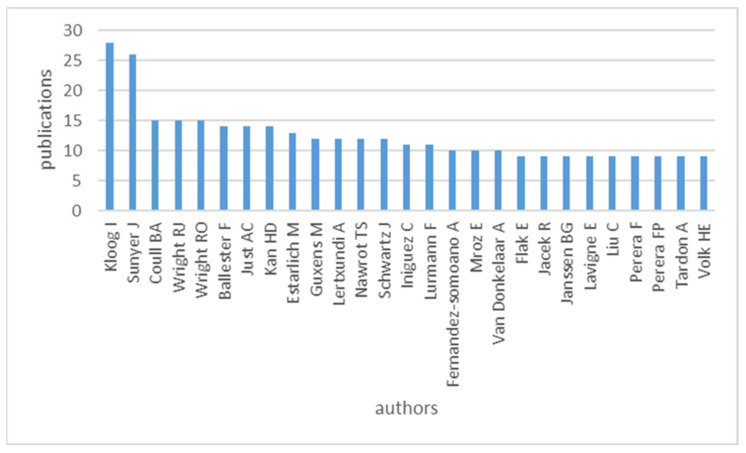
The most productive authors in the prenatal exposure to air pollution literature.

Several authors have contributed to the publications on prenatal exposure to air pollution. Figure 4 shows the top leading authors in this field with Kloog, J. as the most productive author with 28 publications (6.39% fractionalized value), followed by Sunyer, J and Coull, B.A with 26 and 15 papers, respectively. Only the authors with a minimum of nine publications are included in the graph. 

#### 3.1.3. Web of Science Categories

**Figure 5:**
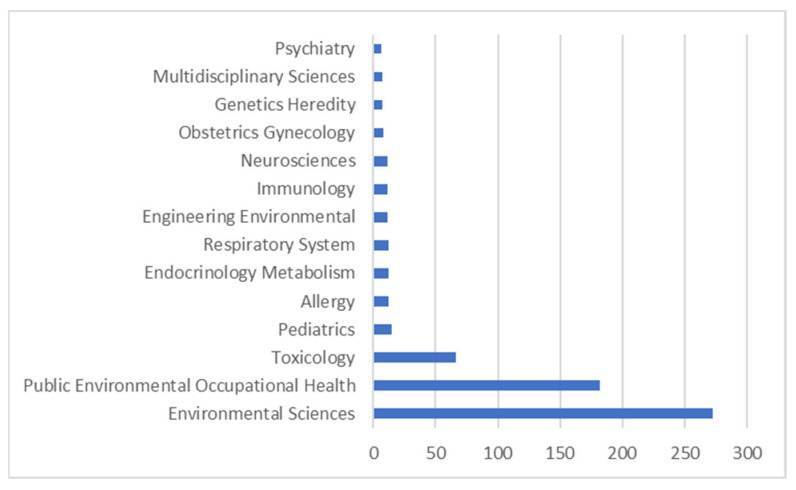
The top 15 disciplines with the highest publications on prenatal exposure to air pollution.

Several WoS categories contributed to publications on prenatal exposure to air pollution; the figure below illustrates the top 15 WoS categories with the highest number of publications. It can be observed that most publications were from environmental science with 273 papers, followed by public health/environment occupational health, toxicology, and pediatrics (Figure 5).

### 3.2. Visualization Maps

VOSViewer is used to execute visualization analyses, including collaborative network analysis, keyword co-occurrence analysis, and reference citation analysis in the field of the prenatal exposure to air pollution. VOSViewer visualization maps consist of nodes that are interconnected by arcs, where the nodes represent items of interest in a map, and the arcs represent the relatedness between nodes connected by such an arc. For instance, on the author collaboration visualization map, nodes refer to the authors, and the arcs connect the authors who have authorship collaborations. The size of the node indicates the prominence of the item on the map, and the thickness of the arc reflects the strength of the relatedness between the connected items (nodes). The bigger the node, the more important the item. On the other hand, the thicker the arc, the higher the strength of relatedness between the connected items [12].

#### 3.2.1. Co-Authorship Analysis

**Figure 6:**
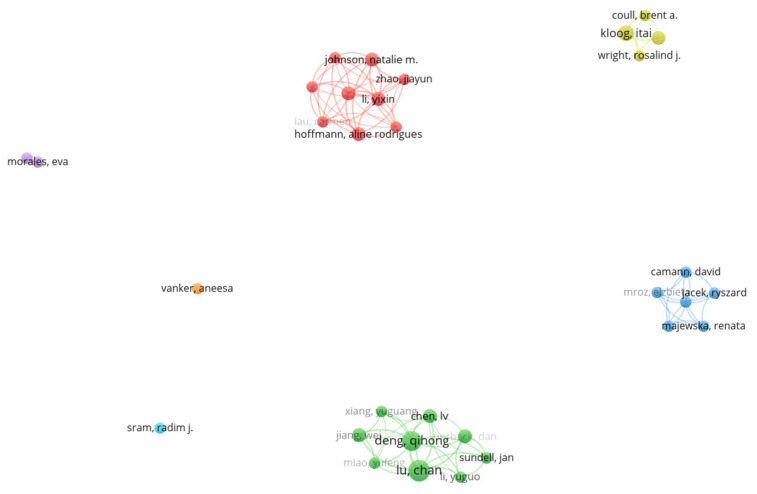
Co-authorship analysis.

Co-authorship analysis depicts authorship collaboration. Author collaboration on prenatal exposure to air pollution publications is shown in the figure below. Only authors with a minimum of two papers were considered. Consequently, only 32 authors met the threshold. Figure 6 shows seven clusters. Authors in the same cluster have high collaboration strength. The red cluster (containing Lau, Carmen) has the highest number of authors, followed by the green cluster (containing Chen, L.V.). It can be observed from Figure 6 that there has been very limited collaboration among authors in the field of prenatal exposure to air pollution. There are two clusters, light blue (Sram Radim, J) and orange clusters (Vanker Annessa), containing one author each. Additionally, clusters are not interconnected. This, therefore, suggests very limited collaboration among the authors in the field of prenatal exposure to air pollution. On the other hand, authors such as Kloog, Itai., Li, Chan, and Deng, Qihong are denoted with bigger circles to indicate their dominance in the publications on prenatal exposure to air pollution. This information concurs with the discussion in Figure 4, where these authors are identified as the most productive authors in this research field. 

#### 3.2.2. Institution Co-Authorship Analysis

**Figure 7:**
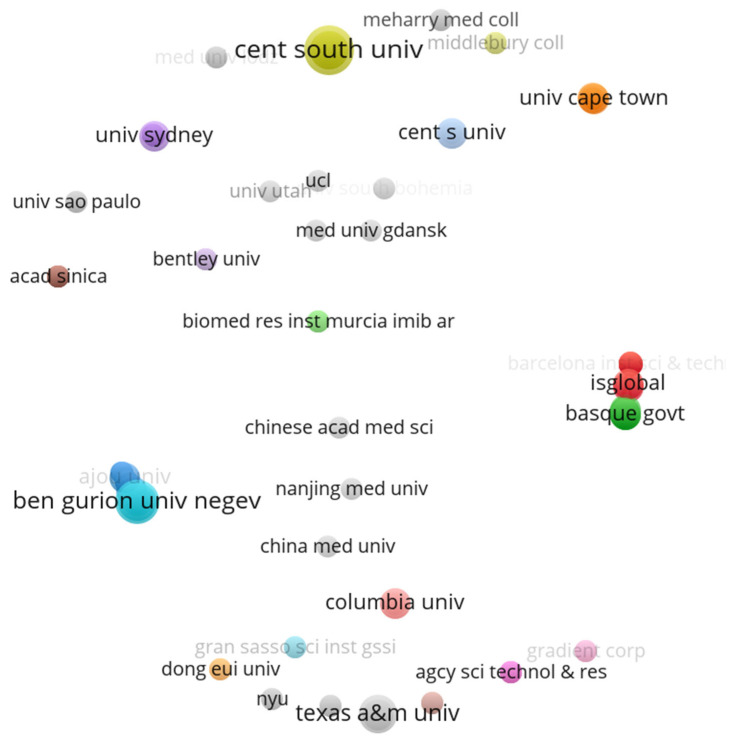
Institution co-authorship map for prenatal exposure to air pollution publications.

Several institutions contributed to the research on prenatal exposure to air pollution. Figure 7 below depicts the institution co-authorship analysis map. Any institution that has contributed to the publication, regardless of the number of papers, was included in the map. The map shows 32 clusters. The cluster with the highest number of items (institutes) is the red cluster, reflecting 25 institutions. There were eight clusters with only one institution each, which indicates that these institutions have not collaborated with any other institute. These institutions include the University of Utah, Shangdong University, the University of Cape Town, and Nanjing Medical University in China. The institution co-authorship map has a structure similar to that of the author co-authorship map, but the map is not interconnected. This is an indication of lack of collaboration among the institutions when it comes to prenatal exposure to air pollution. Nevertheless, Pompeu Fabra University showed the highest collaboration strength of 35, and had has 151 citations, followed by ISGlobal (Barcelona Institute of global health) and the Barcelona Institute of Science and Technology, with collaboration strengths of 31 and 24, respectively. However, these institutions were not the most productive institutes. The most productive institution, denoted by the bigger circle, was Cent South University in China, followed by Ben-gurion University of Negev, Tsinghau University in Israel, and China.

#### 3.2.3. Country/Region Co-Authorship Analysis

**Figure 8:**
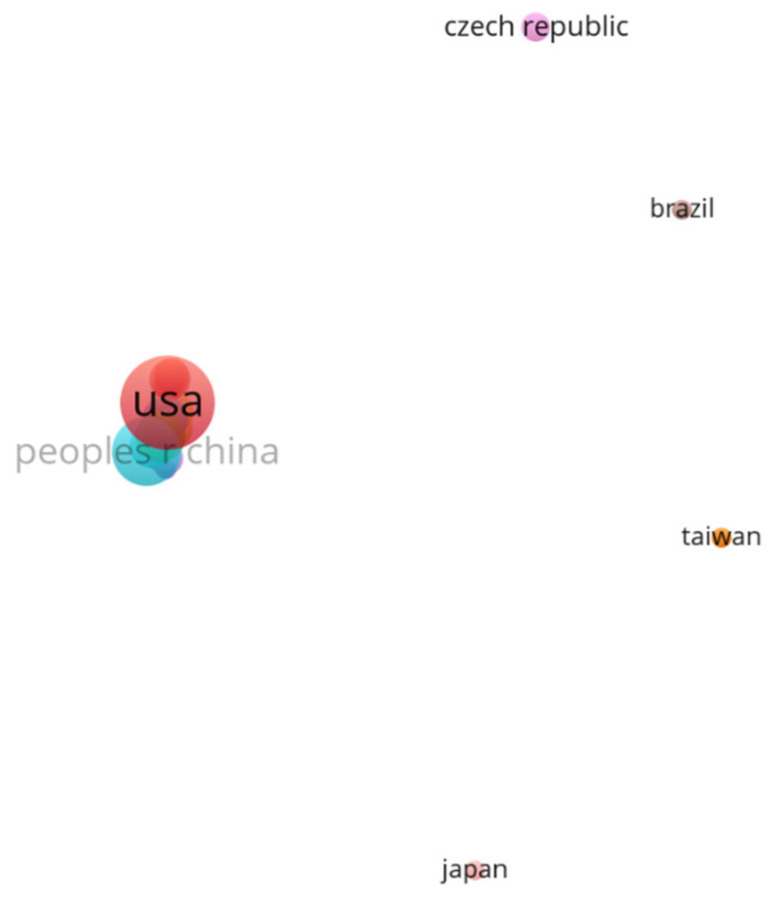
Country/region co-authorship map for prenatal exposure to air pollution publication.

Country co-authorship analysis was carried out to determine collaboration among the countries on prenatal exposure to air pollution. Figure 8 shows the collaboration of 29 countries that have contributed at least one publication in this field. There are ten clusters, the largest being the red clusters, with seven countries. There were three clusters with only one country each and two clusters with only two countries each. It is not surprising to observe that there has also been a lack of collaboration among the countries, as the lack of collaboration was already highlighted among the authors and institutions in the previous sections. Countries such as Brazil and Czech Republic have not collaborated with any countries. 

**Figure 9:**
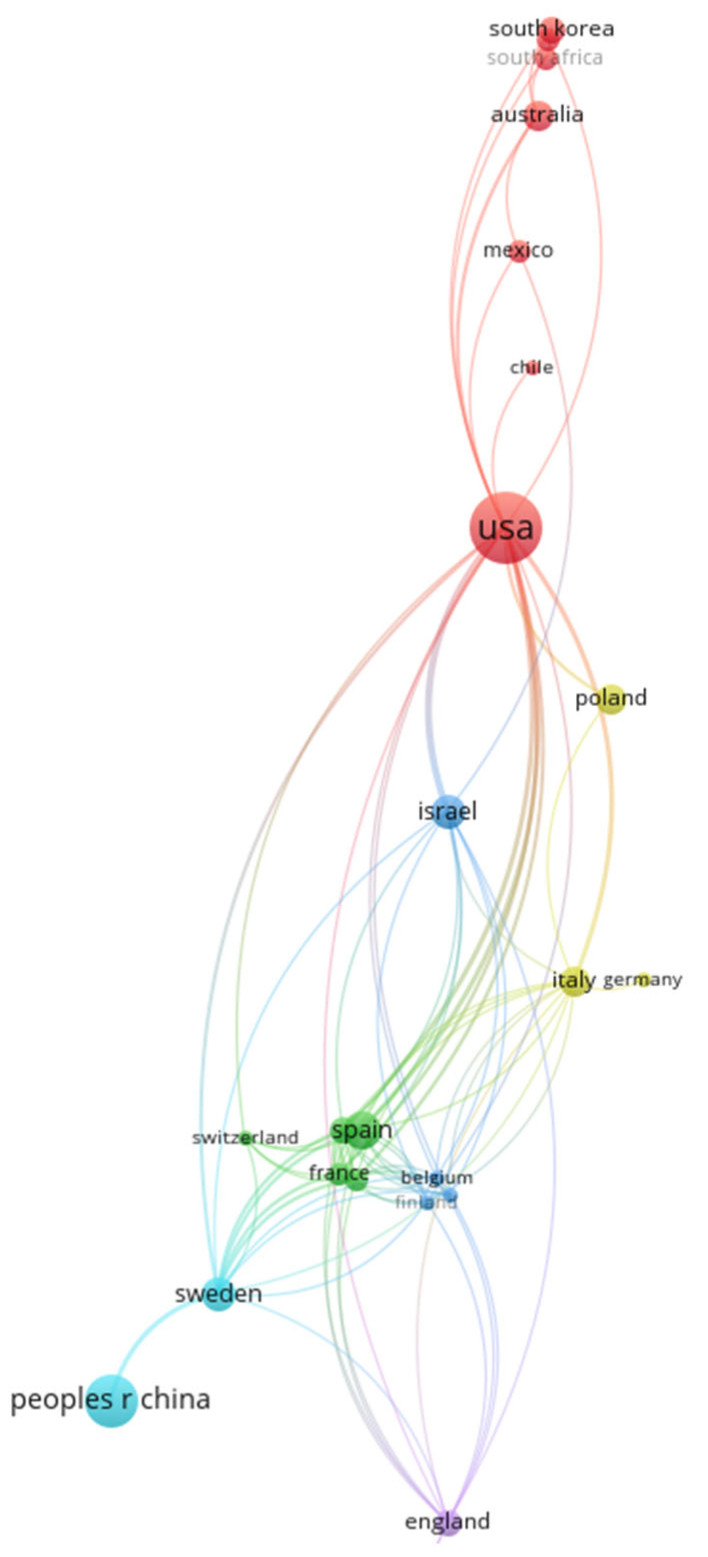
Expanded country/region collaboration map.

The area of the map in Figure 8 depicting collaborating countries/regions was enlarged for visibility purposes and is shown in Figure 9. 

The map in Figure 9, as in Figure 3 (contribution by country/territories), shows the USA, the People’s Republic of China, and Spain as the most productive countries in the literature on prenatal exposure to air pollution. The USA was also the most collaborative country, with a collaboration strength of 34. The USA had strong collaboration with countries such as Australia, Chile, Mexico, South Korea, and South Africa. Spain was also collaborative; it had a collaboration strength of 19 and it mostly collaborated with France. The most productive countries were also collaborative, with the exception of the People’s Republic of China. The People’s Republic of China had collaborated with Sweden only.

#### 3.2.4. Keyword Co-Occurrence Analysis

The results from VOSViewer, which depict the visualization of the keywords co-occurrence analysis, are shown in the figure below. Keyword co-occurrence analysis was used to explore the research trends in prenatal exposure to air pollution. Keywords with a frequency of two or more were considered. Keywords were grouped into seven clusters. The keywords in the same cluster are either keywords strongly related to each other or that frequently appear together in published papers on prenatal exposure to air pollution.

The red cluster has the most elements and is an indication that research on prenatal exposure to air pollution has ample papers with the keywords appearing in this cluster. The dominant keywords appearing in the red cluster include nitric dioxide exposure, symptoms, asthma, pneumonia, parental smoking, respiratory tract infection, parental smoking, and lung function. The cluster with the second-highest number of keywords is green. This cluster contains keywords such as mortality, gene expression, and risk. The cluster with the smallest number of keywords is the orange cluster. It consists of keywords such as atopic dermatitis, prospective birth cohort, and tobacco smoke.

**Figure 10:**
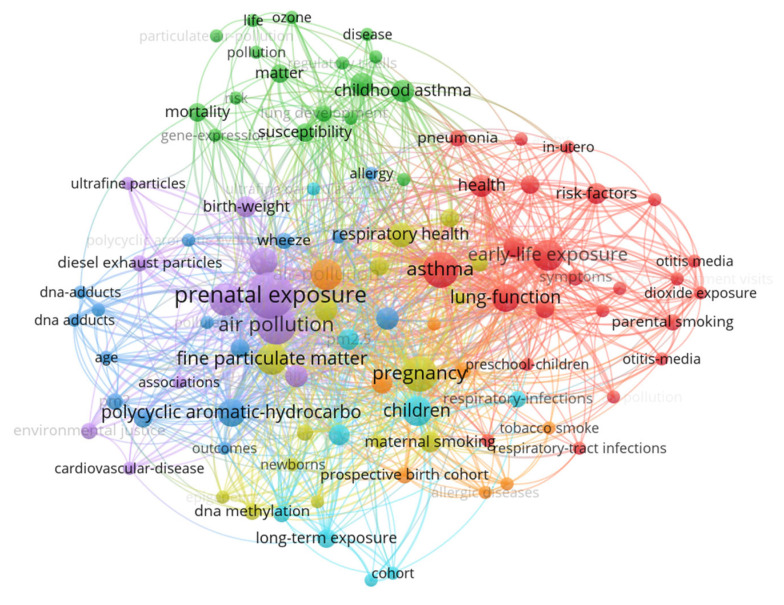
Keyword co-occurrence map.

The keywords with high frequency are denoted with big nodes, including keywords such as air pollution, asthma, lung function, pregnancy, oxidative stress, and fine particle matter. These are the words that have been intensively researched in prenatal exposure to air pollution. On the other hand, keywords represented with smaller nodes indicate keywords that received the least attention from researchers or emerging words. These includes epigenetics, spirometry, congenital anomalies, cardiovascular disease, and tobacco smoking (Figure 10).

The Table 2 below indicates the top 14 keywords with the highest occurrence in the literature of prenatal exposure to air pollution.

#### 3.2.5. Most-Cited Papers in the Prenatal Exposure to Air Pollution Literature

**Figure 11:**
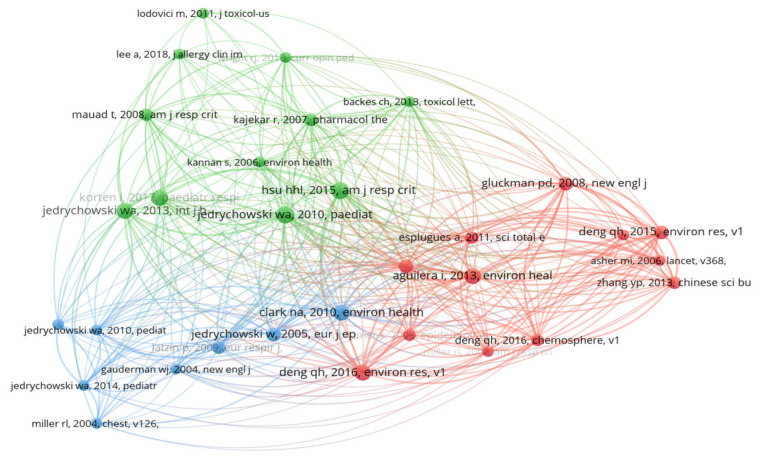
Reference co-citation map.

Reference co-citation determines the number of times two or more papers are cited together and can also be used to reflect the most-cited papers [16]. In this study, reference co-citation was conducted to determine the most-cited papers in prenatal exposure to air pollution literature and papers that address the same issues. This is because the more often two papers are co-cited, the more similar they are. Only papers with the minimum co-citation of 5 were considered, and 31 papers met this threshold. Figure 11 shows the most-cited papers in the prenatal exposure to air pollution literature. The bigger the node that represents the paper, the more cited the paper.

Figure 11 reflects that the most-cited papers were ‘Effect of prenatal exposure to fine particulate matter on ventilatory lung function of preschool children of non-smoking mothers’ by Jedrychowski et al. [17] and ‘Prenatal particulate air pollution and asthma onset in urban children. Identifying sensitive windows and sex differences.’ by Hsu et al. [18]. These papers are represented by the biggest nodes. The top five most-cited papers as per the reference co-citation map (from WoS) are illustrated in Table 3 for readability purposes. All these highly cited papers are journal papers.

## 4. Discussion

The environment is of special importance to the unborn child because of its underdeveloped defenses and rapid neurodevelopment [10]. Air pollution is recognized by the World Health Organization (WHO) as one of the biggest health threats of our time [22]. Air pollution was found to have an association on clinically diagnosed health outcomes [23]. This bibliometric review shows that publications on prenatal exposure to air pollution have increased over the years. However, there has been limited collaboration among authors, higher institutions, and different countries. Additionally, few African authors have published on prenatal exposure to air pollutants.

This study shows that the literature on prenatal exposure to air pollutants increased between 1994 and 2022. The greatest number of articles was published between 2019 and 2022, with the most published in 2021. This could be due to the fact that there is more awareness about preventing certain disease conditions before they manifest later in life. The damage caused by air pollution does not start when an individual is outside of the womb; it usually starts from when he is inside the womb [23]. The country with the highest number of publications was the USA, with 214 publications, making up 48.9% of total publications, followed by China, then Spain. As a developing country, China has a vast geographical area, an enormous population, and various sources of air pollution due to decades of rapid industrial growth, rising domestic energy consumption, and an increase in automobile traffic and other emissions-intensive economic activities [24,25]. Africa is not represented, even though ambient air pollution is increasing in the continent because of increased urbanization [26]. In Africa, the predominant air pollution source is the household, which is already declining, but ambient air pollution is increasing because of industrialization and urbanization [26].

Regarding collaboration among countries for research on prenatal exposure to air pollutants, very few researchers from different countries have collaborated. Collaboration among researchers from different countries is very important to this type of study as this will help to understand the associations between prenatal exposure to air pollution and adverse birth outcomes, especially if standardized parallel analyses in datasets from different countries are used [27]. In 2007, two international workshops of experts on air pollution and adverse pregnancy outcomes were held in Germany and Mexico to look into how collaborations could be enhanced among the researchers working in this area of study and to promote dialogue among epidemiologists, toxicologists, clinicians, and biostatisticians [27]. However, the results of this study show that not much has been achieved with regard to collaboration between countries.

Out of the various health disciplines, it was observed that most publications came from environmental science, followed by public health/environment occupational health, and toxicology, but very few from pediatrics and other disciplines. Different studies have shown that many birth and health outcomes have been linked to prenatal exposure to air pollution; yet, researchers in the different clinical health disciplines are not collaborating with those in the environmental science and public health/environmental and occupational health. In a study conducted by Peterson et al. [28], high exposures to PM_2.5_ and PAH generally disrupted neurotypical brain-behavior associations such as ADHD, anxiety, socialization, and intelligence measures. Additionally, fetal macrosomia has been linked to prenatal exposure to PM_2.5_, NO_2_, and O_3_ [9].

Of the different health outcomes associated with prenatal exposure to air pollution, respiratory symptoms were the most studied judging from the results from the keywords co-occurrence analysis. The dominant keywords are nitric dioxide, exposure, symptoms, asthma, pneumonia, parental smoking, respiratory tract infection, and lung function. More studies need to be conducted on epigenetics, congenital anomalies, cardiovascular diseases, and atopic dermatitis, as well as mortality in relation to prenatal exposure to air pollution.

Furthermore, the most-cited document is a journal article focused on the effect of prenatal exposure to fine particulate matter on the ventilatory function of preschool children of non-smoking smokers (‘Effect of prenatal exposure to fine particulate matter on ventilatory lung function of preschool children of non-smoking mothers’) by Jedrychowski et al. (2010) [17]. Mothers were recruited in either their first or second trimesters of pregnancy, and their children were followed up from newborn to 5 years of age. The study was a collaboration between researchers from two different institutions and countries: Jagiellonian University in Krakow and Columbia University in New York [17]. The study was a properly implemented birth cohort study. The use of a birth cohort study allowed for the determination of the causal relationship between potential risk factors during the prenatal or postnatal period and the health status of individuals from newborn up to childhood and potentially into adulthood [29]. This might have made the study highly influential and trustworthy among researchers. Methodology, study design, international collaboration of authors, and accessibility are among the factors that have been linked to the frequent citation of a journal article [30]. The study may be an open-access article, making it to be accessible to every researcher. Another reason for the high number of citations could be prior publication by the authors [31], especially Jedrychowski, who had previously published research articles [32,33,34] on exposure to air pollution and its health outcomes.

This study provides a basic overview of global research on prenatal exposure to air pollution. It highlights the scarcity of studies in some regions of the world as well as collaboration between researchers in different countries and institutions. Additionally, air pollutant and disease-specific bibliometric analyses can be carried out to disseminate information that has been unexplored in the field related to prenatal exposure to air pollution.

The current study is not without its limitations. The reviewed documents were from 1994 to 2022, and a total of 959 were identified. Some of the documents were not relevant for the study, leaving our study database with 438 documents. Articles written in English and other languages were included in the analysis. However, only seven documents were in a non-English language.

## 5. Conclusions

Based on the trend in the research on prenatal exposure to air pollution, more collaboration should be encouraged between researchers from different countries and disciplines. More countries in Africa should also engage in this research field as this prevents reliance on the results of studies from other continents.

## Figures and Tables

**Table 1:** Types and number of documents in prenatal exposure to air pollution literature.

Document Types	Record Count	% of 438
Article	365	83.333
Review Article	50	11.416
Meeting Abstract	17	3.881
Early Access	5	1.142
Editorial Material	2	0.457
Letter	2	0.457
Proceeding Paper	2	0.457
Book Chapters	1	0.228
Correction	1	0.228

**Table 2:** Top 14 keywords with the highest frequency in prenatal exposure to air pollution literature.

Rank	Keyword	Frequency	Rank	Keyword	Frequency
1	Air pollution	29	8	Children	10
2	Prenatal exposure	25	9	Polycyclic aromatic hydrocarbons	9
3	Pregnancy	15	10	Lung function	9
4	Asthma	15	11	Oxidative stress	9
5	Particulate matter	12	12	Respiratory health	7
6	Early life exposure	11	13	Respiratory symptoms	6
7	Fine particular matter	11	14	Association	6

**Table 3:** The top five most-cited papers as perWoS.

Rank	Authors and Year	Paper Title	Paper Type	Citations from WoS
1	Jedrychowski, W.A., Perera, F.P., Maugeri, U., Mroz, E., Klimaszewska-Rembiasz, M., Flak, E., Edwards, S. and Spengler, J.D., 2010. [17]	Effect of prenatal exposure to fine particulate matter on ventilatory lung function of preschool children of non-smoking mothers	Journal	13
2	Leon Hsu, H.H., Mathilda Chiu, Y.H., Coull, B.A., Kloog, I., Schwartz, J., Lee, A., Wright, R.O. and Wright, R.J., 2015. [18]	Prenatal particulate air pollution and asthma onset in urban children. Identifying sensitive windows and sex differences.	Journal	13
3	Clark, N.A., Demers, P.A., Karr, C.J., Koehoorn, M., Lencar, C., Tamburic, L. and Brauer, M., 2010. [19]	Effect of early life exposure to air pollution on development of childhood asthma.	Journal	11
4	Aguilera, I., Pedersen, M., Garcia-Esteban, R., Ballester, F., Basterrechea, M., Esplugues, A., Fernández-Somoano, A., Lertxundi, A., Tardón, A. and Sunyer, J., 2013. [20]	Early life exposure to outdoor air pollution and respiratory health, ear infections, and eczema in infants from the INMA study	Journal	11
5	Deng, Q., Lu, C., Li, Y., Sundell, J. and Norbäck, D., 2016. [21]	Exposure to outdoor air pollution during trimesters of pregnancy and childhood asthma, allergic rhinitis, and eczema	Journal	11

## References

[1] Sun J., Zhou Z., Huang J., Li G. (2020). A Bibliometric Analysis of the Impacts of Air Pollution on Children. Int. J. Environ. Res. Public Health.

[2] Health Effects Institute (2019). State of Global Air 2019. Special Report.

[3] Su X., Zhang S., Lin Q., Wu Y., Yang Y., Yu H., Huang S., Luo W., Wang X., Lin H. (2021). Prenatal exposure to air pollution and neurodevelopmental delay in children: A birth cohort study in Foshan, China. Sci. Total. Environ..

[4] Uwak I., Olson N., Fuentes A., Moriarty M., Pulczinski J., Lam J., Xu X., Taylor B.D., Taiwo S., Koehler K. (2021). Application of the navigation guide systematic review methodology to evaluate prenatal exposure to particulate matter air pollution and infant birth weight. Environ. Int..

[5] Vieira S. (2015). The health burden of pollution: The impact of prenatal exposure to air pollutants. Int. J. Chronic Obstr. Pulm. Dis..

[6] Yen H.-C., Lin C.-H., Lin M.-C., Hsu Y.-C., Lin Y.-H. (2022). Prenatal Exposure to Air Pollution and Immune Thrombocytopenia: A Nationwide Population-Based Cohort Study. Front. Pediatr..

[7] Deng S.-Z., Jalaludin B.B., Antó J.M., Hess J.J., Huang C.-R. (2020). Climate change, air pollution, and allergic respiratory diseases: A call to action for health professionals. Chin. Med. J..

[8] Kim J.B., Prunicki M., Haddad F., Dant C., Sampath V., Patel R., Smith E., Akdis C., Balmes J., Snyder M.P. (2020). Cumulative Lifetime Burden of Cardiovascular Disease From Early Exposure to Air Pollution. J. Am. Heart Assoc..

[9] Shang L., Yang L., Yang W., Xie G., Wang R., Sun L., Xu M., Zhang B., Li J., Yue J. (2021). Prenatal exposure to air pollution and the risk of macrosomia: Identifying windows of susceptibility. Sci. Total. Environ..

[10] Oudin A., Frondelius K., Haglund N., Källén K., Forsberg B., Gustafsson P., Malmqvist E. (2019). Prenatal exposure to air pollution as a potential risk factor for autism and ADHD. Environ. Int..

[11] Morgan Z.E.M., Bailey M.J., Trifonova D.I., Naik N.C., Patterson W.B., Lurmann F.W., Chang H.H., Peterson B.S., Goran M.I., Alderete T.L. (2023). Prenatal exposure to ambient air pollution is associated with neurodevelopmental outcomes at 2 years of age. Environ. Health.

[12] Van Eck N.J., Waltman L. (2010). Software survey: VOSviewer, a computer program for bibliometric mapping. Scientometrics.

[13] van Eck N.J., Waltman L. (2017). Citation-based clustering of publications using CitNetExplorer and VOSviewer. Scientometrics.

[14] Yang W., Zhang J., Ma R. (2020). The Prediction of Infectious Diseases: A Bibliometric Analysis. Int. J. Environ. Res. Public Health.

[15] Yu Y., Li Y., Zhang Z., Gu Z., Zhong H., Zha Q., Yang L., Zhu C., Chen E. (2020). A bibliometric analysis using VOSviewer of publications on COVID-19. Ann. Transl. Med..

[16] Li J., Goerlandt F., Reniers G. (2020). Trevor Kletz’s scholarly legacy: A co-citation analysis. J. Loss Prev. Process. Ind..

[17] Jedrychowski W.A., Perera F.P., Maugeri U., Mroz E., Klimaszewska-Rembiasz M., Flak E., Edwards S., Spengler J.D. (2010). Effect of prenatal exposure to fine particulate matter on ventilatory lung function of preschool children of non-smoking mothers. Paediatr. Périnat. Epidemiol..

[18] Hsu H.-H.L., Chiu Y.-H.M., Coull B.A., Kloog I., Schwartz J., Lee A., Wright R.O., Wright R.J. (2015). Prenatal Particulate Air Pollution and Asthma Onset in Urban Children. Identifying Sensitive Windows and Sex Differences. Am. J. Respir. Crit. Care Med..

[19] Clark N.A., Demers P.A., Karr C.J., Koehoorn M., Lencar C., Tamburic L., Brauer M. (2010). Effect of early life exposure to air pollution on development of childhood asthma. Environ. Health Perspect..

[20] Aguilera I., Pedersen M., Garcia-Esteban R., Ballester F., Basterrechea M., Esplugues A., Fernández-Somoano A., Lertxundi A., Tardón A., Sunyer J. (2013). Early life exposure to outdoor air pollution and respiratory health, ear infections, and eczema in infants from the INMA study. Environ. Health Perspect..

[21] Deng Q., Lu C., Li Y., Sundell J., Norbäck D. (2016). Exposure to outdoor air pollution during trimesters of pregnancy and childhood asthma, allergic rhinitis, and eczema. Environ. Res..

[22] Cohen A.J., Brauer M., Burnett R., Anderson H.R., Frostad J., Estep K., Balakrishnan K., Brunekreef B., Dandona L., Dandona R. (2017). Estimates and 25-year trends of the global burden of disease attributable to ambient air pollution: An analysis of data from the Global Burden of Diseases Study 2015. Lancet.

[23] Ritz B., Liew Z., Yan Q., Cuia X., Virk J., Ketzel M., Raaschou-Nielsen O. (2018). Air pollution and autism in Denmark. Environ. Epidemiology.

[24] Yang F., Jia C., Yang H., Yang X. (2022). Development, hotspots and trend directions of groundwater salinization research in both coastal and inland areas: A bibliometric and visualization analysis from 1970 to 2021. Environ. Sci. Pollut. Res..

[25] Fisher S., Bellinger D.C., Cropper M.L., Kumar P., Binagwaho A., Koudenoukpo J.B., Park Y., Taghian G., Landrigan P.J. (2021). Air pollution and development in Africa: Impacts on health, the economy, and human capital. Lancet Planet. Health.

[26] Woodruff T.J., Parker J.D., Adams K., Bell M.L., Gehring U., Glinianaia S., Ha E.-H., Jalaludin B., Slama R. (2010). International Collaboration on Air Pollution and Pregnancy Outcomes (ICAPPO). Int. J. Environ. Res. Public Health.

[27] Slama R., Darrow L., Parker J., Woodruff T.J., Strickland M., Nieuwenhuijsen M., Glinianaia S., Hoggatt K.J., Kannan S., Hurley F. (2008). Meeting Report: Atmospheric Pollution and Human Reproduction. Environ. Health Perspect..

[28] Peterson B.S., Bansal R., Sawardekar S., Nati C., Elgabalawy E.R., Hoepner L.A., Garcia W., Hao X., Margolis A., Perera F. (2022). Prenatal exposure to air pollution is associated with altered brain structure, function, and metabolism in childhood. J. Child Psychol. Psychiatry.

[29] Canova C., Cantarutti A. (2020). Population-Based Birth Cohort Studies in Epidemiology. Int. J. Environ. Res. Public Health.

[30] Tahamtan I., Afshar A.S., Ahamdzadeh K. (2016). Factors affecting number of citations: A comprehensive review of the literature. Scientometrics.

[31] Repiso R., Moreno-Delgado A., Aguaded I. (2020). Factors affecting the frequency of citation of an article. Iberoam. J. Sci. Meas. Commun..

[32] Jedrychowski W., Whyatt R.M., Camann D.E., Bawle U.V., Peki K., Spengler J.D., Dumyahn T.S., Penar A., Perera F.F. (2003). Effect of prenatal PAH exposure on birth outcomes and neurocognitive development in a cohort of newborns in Poland. Study design and preliminary ambient data. Int. J. Occup. Med. Environ. Health.

[33] Jedrychowski U.M.W. (2001). Transient or persistent asthma-like symptoms and lung growth over 2-year follow-up in pre-adolescent children. J. Epidemiol. Biostat..

[34] Jedrychowski W., Becher H., Wahrendorf J., Basa-Cierpialek Z. (1990). A case-control study of lung cancer with special reference to the effect of air pollution in Poland. J. Epidemiology Community Health.

